# Using Multiverse Analysis to Highlight Differences in Convergent Correlation Outcomes Due to Data Analytical and Study Design Choices

**DOI:** 10.1177/10731911221127904

**Published:** 2022-09-29

**Authors:** Sam S. Webb, Nele Demeyere

**Affiliations:** 1 University of Oxford, UK

**Keywords:** multiverse analysis, specification curve analysis, executive function, validation, clinical population

## Abstract

In neuropsychological research, there are a near limitless number of different approaches researchers can choose when designing studies. Here we showcase the multiverse/specification curve technique to establish the robustness of analytical pathways choices within classic psychometric test validation in an example test of executive function. We examined the impact of choices regarding sample groups, sample sizes, test metrics, and covariate inclusions on convergent validation correlations between tests of executive function. Data were available for 87 neurologically healthy adults and 117 stroke survivors, and a total of 2,220 different analyses were run in a multiverse analysis. We found that the type of sample group, sample size, and test metric used for analyses affected validation outcomes. Covariate inclusion choices did not affect the observed coefficients in our analyses. The present analysis demonstrates the importance of carefully justifying every aspect of a psychometric test validation study a priori with theoretical and statistical factors in mind. It is essential to thoroughly consider the purpose and use of a new tool when designing validation studies.

Depending on the way in which neuropsychological data are collected, processed, or scored, there are potentially hundreds of final data sets that could be compiled, which may lead down different analytical paths to different findings (Silberzahn et al., 2018). Steegen et al. (2016) modeled how to account for choices regarding data processing, cleaning, and analysis, through specification curve/multiverse analysis (see Simonsohn et al., 2020 for a general introduction), now referred to as *multiverse analysis*. Multiverse analysis is a statistical technique that allows researchers to view the impact of analysis choices on statistical outcomes, both graphically and via inferential statistics (Simonsohn et al., 2020). If a researcher has an a priori prediction of a particular statistical outcome, running multiple versions of that statistical analysis, adapting for factors such as sample size, covariate inclusion, outlier exclusion and other cleaning techniques, can aid in determining the robustness of their finding (Steegen et al., 2016). For instance, if in most iterations of the analysis, the statistical outcomes are non-significant, assuming all the factors in the multiverse analysis were reasonable, then it casts doubt on the replicability or fidelity of the finding.

The multiverse analysis technique is a variant of existing techniques, such as sensitivity analysis (Pichery, 2014) or robustness analysis (Rosenhead, 2013). These techniques each take decisions that are drawn from uncertainty and examine the impact of these on final models or decisions (Hall et al., 2022). There are a variety of mathematical, statistical, and or graphical versions of sensitivity analysis (Frey & Patil, 2002). The multiverse analysis technique is designed to address the variance in hypothesis testing outcomes and includes a statistical test of the multiverse analysis outcome to a null distribution (Simonsohn et al., 2020). In doing this, the multiverse is a superior analysis technique to assess the impact of analytical choices on the outcomes of psychometric assessment as it provides researchers with statistics to infer whether the analysis on the whole shows robust or inconsistent outcomes.

Here, we propose and demonstrate a pathway to apply this technique to convergent validation analysis and provide an example of tests of executive function. In this article, *validation* refers to the process of establishing evidence for the interpretation of tests used in their intended populations/settings etc., and *validation outcomes* refer to the hypothesized and intended outcomes of a study (e.g., that there will be a strong positive correlation between two scales). In this article, we use an example of establishing convergent validity in tests of executive functioning (see Goldstein et al., 2014 for an overview capturing the complexity of defining the term). Currently, the multiverse technique is not widely used as a newly developed technique (Simonsohn et al., 2020), but it is picking up traction regarding its use in psychological research. Limited research is published with neuropsychological data, except in the case of Moore & Demeyere, 2022, which employs the statistical techniques and code used in this article, though applied to a question of the robustness of regression analyses. We anticipate future research to use this technique more widely, supported by the openly available code.

The focus of this article is on convergent validity as one of the most common methods to investigate the validity of neuropsychological assessment in general (Pawlowski et al., 2013), and particularly so with regards to the example of a Multiple Errands Tasks we will present here (Webb et al., 2021). The 2014 Standards for Educational and Psychological Testing (Standards 1.12 and1.16) state that if a cognitive process is being assessed via a test, in this case executive functioning, then evidence must be generated that the test does assess that cognitive process (American Educational Research Association et al., 2014), and to generate evidence using well construct-validated tools. We note that best practice for a full validation requires multiple examining the different aspect of the validity of the test and test interpretation (Kane, 2013), though we have limited this multiverse to construct validation.

A 2013 systematic review of validation procedures for neuropsychological batteries aimed at clinical populations, examined 147 studies that were published between 2005 and 2012 (Pawlowski et al., 2013). The clinical populations in the reviewed studies predominantly included dementia and Alzheimer’s disease, mild cognitive impairment, and acquired brain injury, though other clinical groups were also present. In terms of sample group used, the systematic review found that half of the studies included both healthy controls and a clinical group, followed by 35.40% that included just clinical groups, and 13.50% that included just healthy controls (Pawlowski et al., 2013). Sample sizes ranged from 13 to 705 people for clinical groups and three to 13,000 healthy controls, with the majority of papers having fewer than 100 participants per group (Pawlowski et al., 2013).

Sample group and size should be pre-defined depending on the intended target group for the test (e.g., if the test is developed for diagnosing dementia, it is appropriate to validate the test on those with dementia), and whether it is used to determine impairment compared with normative cut-offs (e.g., generating validity evidence from healthy control participants and using the normative cut-offs to diagnose impairment in a clinical group) (American Educational Research Association et al., 2014). Sample size is often arbitrary in clinical research with no clear a priori justification (Anthoine et al., 2014), and sample size justifications differ dependent on the type of validity evidence being collected (e.g., factor analysis to investigate internal factor structure of a scale versus regressions for predicting outcomes; Hobart et al., 2012). Sample size affects validation outcomes because the power to detect effects will be drastically reduced the smaller the sample is (Cohen, 1992a; Jones et al., 2003), and additionally small samples could reveal a large correlation given a less reliable estimate of the correlation (Schönbrodt & Perugini, 2013).

When examining whether or not a test measures a construct, or cognitive process, it is important to acknowledge a priori in which test metric is used and for what purpose (American Educational Research Association et al., 2014). When assessing tests of executive functioning, researchers should specify which component of executive function is to be tested or whether multiple processes are assessed (Miyake et al., 2000) Specific metrics (i.e., overall score, subscore, etc.) should be carefully selected for purpose and use and validity evidence collected separately.

The Trail Making Test (TMT) (Reitan, 1971), as adapted for the shape-based analogue Oxford Cognitive Screen–Plus (OCS-Plus) version (Demeyere et al., 2021), is a popular test of executive function (Lin et al., 2021) and provides a good example where one test has multiple outcome metrics. The original TMT (Reitan, 1971) required connecting the letters (Part A) or connecting numbers and letters in ascending order (Part B) as fast as possible without lifting the pen and without making any errors (Specka et al., 2022), and used time and errors made. Further scores can also be derived from the test, including a ratio of B/A errors or time (Arbuthnott & Frank, 2000; Reitan, 1971), and a subtraction of B−A time or errors (Heaton et al., 1985), or even B−A/A (Sánchez-Cubillo et al., 2009) among others. The ratio of B/A is thought of as a purer measure of executive functioning, removing variance which is attributable to visual scanning or motor speed measured in part A (Specka et al., 2022). The OCS-Plus version uses a ratio score of mixed over baseline trails, and also a processing measure accounting for both accuracy and time taken on the baseline trails.

In practice, authors do not often justify the choice of a metric explicitly, by discussing either the psychometrics of the score or the intended use of the metric (Kopp et al., 2015). Typically, researchers refer to previous studies or clinical practice, although research may mention that there are alternative metrics (Arbuthnott & Frank, 2000). Metric justification is particularly important where different metrics differentially relate to assessments of the same construct that the TMT measures (Sánchez-Cubillo et al., 2009). For example, Arbuthnott and Frank (2000) found that subtraction scores from Trail B and Trail A did not relate to reaction time on a switching paradigm, but ratio scores did. The use of a different metric, in this case, affected whether or not the task would be considered convergent or not with a switching construct, illustrating the importance of choosing measures carefully a priori with justification from theoretical expectations and psychometric/intended use.

Norms for tests of executive function are often stratified by age and education (Tombaugh, 2004) due to the known influence of these variables on cognitive performance. Similarly, the inclusion of known covariates is important and common in neuropsychological assessments (Kabuba et al., 2018; cf. Lowe & Reynolds, 1999). Importantly, not including age or education in neuropsychological test validation analyses can incorrectly inflate the correlation coefficients observed as they are both related to performance on the tests, in other words, the inclusion of known covariates will appropriately attenuate correlations, affecting validation outcomes.

In the present multiverse demonstration, we, therefore, chose to focus on (a) sample group used, (b) sample size, (c) metrics used for outcome measures, and (d) covariate inclusion while acknowledging there are many potential other aspects that could affect validation outcomes. We use data from a recently developed multiple errands-based test, the Oxford Digital Multiple Errands Test (OxMET; Webb et al., 2021), which involved comparing the OxMET to two executive functioning tests (i.e., TMT and rule finding test), taken from the recently validated OCS-Plus (see Demeyere et al., 2021 for healthy control validation, and Webb et al., 2022 for subacute and chronic stroke validation).

## Method

We report how we determined our sample size, all data exclusions, all manipulations, and all measures in the study.

### Ethics

This project represents a retrospective secondary analysis of existing data collected as part of two overarching research projects on neurologically healthy aging control participants (Oxford University ethics reference “MSD-IDREC-C1-2013-209”) and data collected within the Oxford Cognitive Screening (OCS) program (Demeyere et al., 2015), which recruited a consecutive sample of stroke survivors during acute hospitalization and conducted follow up neuropsychological assessments at six-months. The OCS program received NHS ethics approval (OCS-Tablet and OCS-Recovery studies, NHS RECs 14/LO/0648 and 18/SC/0044), and all patients provided written or witnessed informed consent.

### Participants

The neurologically healthy adult participants were recruited through existing research volunteer databases, drawing on relatives of stroke survivors, volunteers recruited through our website and center open days, or those who self-referred to the group. Participants were included if they had no self-reported neurological diagnoses or symptoms. For the stroke survivors, no selection criteria regarding specific behavior or lesion characteristics were used with the only two exclusion criteria an inability to stay alert for the duration of testing and incapacity to provide informed consent.

### Materials

We used data from the OxMET psychometric investigation (Webb et al., 2021). The OxMET, described in detail in Webb et al. (2021), is a simplified shopping scenario computer–tablet-based errands task. A single score on overall accuracy is the primary outcome, though full performance metrics including detailed error related data are also calculated automatically and stored in the task. These include the number of rules broken, the frequency of rule breaks, perseverative errors, commissions, omissions, and total errors (Webb et al., 2021).

The tests used to establish convergent validity in the original validation (Webb et al., 2021) were the shape-analogue TMT and the Rule Finding Test from the OCS-Plus (Demeyere et al., 2015). To keep participant numbers consistent in this exemplar, we only use the OxMET, and OCS-Plus tasks.

The OCS-Plus Trails uses a ratio score of the baseline Trail A test trials (both a circle and square condition, connecting small to large circles and large to small squares, respectively) and Trail B (alternating between large squares and small circles), where an executive score is Trail B accuracy / (Trail A circles + Trail A squares) as a percentage. The OCS-Plus further includes “thinking time,” which is the time elapsed from the start of a trial to the first pen stroke. The rule-finding task from the OCS-Plus involves participants predicting the next placement of a red dot which moves around the shapes and changes consecutively without notice between five placement rules. The task uses accuracy and number of rules learned, as well as time to completion as outcome metrics. The test is based on the Brixton Spatial Anticipation test (Burgess & Shallice, 1997), which uses incorrect guesses/errors as the main metric.

### Identification of Specifications

For each test, different metrics were identified from the literature and test manuals, which were conceivable for inclusion in a convergent validity analysis, in addition to theoretically plausible metrics. Each of the plausible metrics in the current project is detailed in Table 1, split by time, accuracy, error, and composite/ratio parameters. As discussed, metrics chosen can depend on the purpose of the test, however, given the many potential choices, it is likely that a variation in choices would occur by different researchers doing the same study (Silberzahn et al., 2018).

**Table 1.** Analysis Specifications From Three Tests of Executive Function Divided Into Accuracy, Error, Time, or Composite/Ratio Scores.

Task	Parameter	Outcome measure
OxMET	Time	Time to completion
	Accuracy	Total accuracy on shopping within rule constraints
	Error	Number of rules broken (out of three)
		Frequency of rules broken
		Task omissions (non-attempted tasks)
		Partial task omissions (incomplete tasks)
		Perseverative errors
		Task commissions (wrong items bought)
		Total errors (rule breaks + omissions + commissions)
	Composite or ratios	Accuracy divided by time to completion
OCS-Plus Trail Making Test	Time	Time to completion of Trail A circles
		Thinking time before starting Trail A circles
		Time to completion of Trail A squares
		Thinking time before starting Trail A squares
		Time to completion of Trail B
		Thinking time before starting Trail B
		Mean time to completion of Trail A
		Mean thinking time of Trail A
	Accuracy	Trail A circles connections accuracy
		Trail A squares connections accuracy
		Trail B connections accuracy
		Mean Trail A connections accuracy
	Error	Errors on Trail A circles
		Errors on Trail A squares
		Errors on Trail B
		Mean error on Trail A
	Composite or ratios	Trail B—Trail A circles
		Trail B—Trail A squares
		Trail B—Mean Trail A
		Trail B—sum of Trail A
		Trail B / Trail A circles
		Trail B / Trail A squares
		Trail B / Mean Trail A
		Trail B / Median Trail A
		Trail B / sum of Trail A
		Trail B / Time to completion Trail B
		Trail A circles accuracy / Time to completion Trail A circles
		Trail A squares accuracy / Time to completion Trail A squares
		Trail A circles errors / Time to completion Trail A circles
		Trail A squares errors / Time to completion Trail A squares
		Trail B errors / Time to completion Trail A squares
OCS-Plus Rule Finding and Switching Task	Time	Time to completion
	Accuracy	Total accuracy
		Number of rules learned
	Error	Total number of errors
	Composite or ratios	Total accuracy /Time to completion
		Total number of errors /Time to completion
		Number of rules learned /Time to completion

*Note.* Trail A refers to the baseline condition on the Trails test from the OCS-Plus. Trail B refers to a switching condition of alternating between circles and squares in the Trails test from the OCS-Plus. All specific OxMET errors are detailed in (Webb et al., 2021). OxMET = Oxford Digital Multiple Errands Test; OCS-Plus = Oxford Cognitive Screen–Plus.

Plausibility of metric choice or analysis depends on many factors (Del Giudice & Gangestad, 2021) but can be summarized as whether the metric would actually be used in an analysis testing a specific hypothesis in a single data set. The aim of running the multiverse is to check the robustness of results answering the same question, so completely arbitrary metric choices in analysis would deflate or inflate the observed effects as it would not be a valid way to answer the same research question. For illustration, when looking at convergent validity of executive function tests a researcher would not plausibly include a metric of the number of cats owned by each participant as a covariate in analysis, as this is not theoretically or statistically justified. In the same way, if investigating the speed of reaction times on executive function tests, only speed should be used and not another metric of pure accuracy or errors, etc. if speed is specific to the hypothesis. Speed could be defined in multiple ways and taken from multiple parts of the same test (e.g., from all blocks of an experiment or just the last block, etc.). Note the definition of theoretically justified or valid choices is debatable (see Del Giudice & Gangestad, 2021, for an excellent discussion and possible solution).

### Planned Analysis

When comparing the OxMET (i.e., totaling 10 different outcome variables) to the Trails (i.e., 30 variables) and Rule finding (i.e., 7) metrics, across inclusion or exclusion of covariates (i.e., 2 conditions of with or without covariates), and across healthy adults, stroke survivors, or both combined, there are a total of 2,220 possible analyses that could be conducted on the data.

We ran Spearman’s Rho correlation and partial correlations for covariates, collecting outcomes of uncorrected *p*-values, 95% confidence intervals, and Spearman’s Rho effect size per 2,220 analyses. Note that correlations are justified here to establish the relationship and associations between tests for validity evidence for convergence of constructs (Krabbe, 2017).

Following Simonsohn et al. (2020), the *p*-value for the significance of a difference between medians from the observed and null effects multiverse was extracted by calculating the proportion of the 500 permutated null analyses, which had a test statistic that was the exact same median value or greater, as in the observed data, and then dividing that by two (significant if below .<05 or if no null values are equal or greater we report *p* <.002). For this median comparison, we rounded median coefficients to two decimal places to maximize the chance of matching medians rather than inflating the risk of non-matching medians if they are >.001 decimal place out.

We also examined the difference in significance levels across the observed effects and null effects models by calculating the proportion of significant results in the null and observed effects models. In this way, we were able to statistically compare the curves of real and null effects by looking at the median coefficient and also the rate of significant findings in both multiverses. After illustrating the output of the multiverse analysis, we compared multiverse outputs first by which sample was used (e.g., healthy adults only, stroke survivors only, or combined sample), then by the size of the samples in each analysis, then by outcome categories of specifications (e.g., accuracy, time, error, composite/ratio scores), and finally by inclusion or exclusion of covariates.

All final analyses were conducted in R studio version 4.1.0, (R Core Team, 2019) using packages *knitr* (Xie, 2014, 2015, 2019) *dplyr* (Wickham, François, et al., 2019), *ggplot2* (Wickham, 2016), *cowplot* (Wilke, 2020), *esc* (Lüdecke, 2019), *remotes* (Hester et al., 2020), *rbbt* (Dunnington & Wiernik, 2021), *Hmisc* (Harrell, 2021), and *rstatix* (Kassambara, 2021). Generation of the multiverse data and graphs used the following additional libraries: *ggm* (Marchetti et al., 2020), *ppcor* (Kim, 2015), *DescTools* (Signorell et al., 2021), *tidyverse* (Wickham, Averick, et al., 2019). The data for all analyses presented here are openly available at DOI 10.17605/OSF.IO/QHKJ2, and adapted from Orben & Przybylski (2019). We present steps to run multiverse analysis following steps described in Simonsohn et al. (2020) in supplementary materials.

## Results

### Participants

In total, 205 participants were included in the analyses (healthy adults = 88, stroke survivors = 117). All stroke survivors had a clinically confirmed diagnosis of stroke. Laterality of stroke; 6.38% bilateral 45.74% left, and 46.81% were right hemisphere. The average time in years between stroke and assessment date was 1.66 (*SD*=1.49, range= 0.01–7.21). Table 2 details the demographic information for the sample.

**Table 2.** Characteristics of the Sample Split by Healthy Adult Group and Stroke Survivor Group.

Demographic	Healthy adults	Stroke survivors	Combined sample
Age (*M, SD*, range)	66.69, 11.67, 21–93	72.4, 12.47, 28–92.87	69.96, 12.43, 21–93
Education (*M, SD*, range)	15.56, 3.63, 6–26	12.89, 3.51, 6–26	14.06, 3.79, 6–26
Handedness (R: L)	78:08	103:11	181:19
Sex (M: F)	42:45	70:47	112:92
Ethnicity (White British: Other)	72:03	76:04	148:07

*Note.* Age is formatted as follows: *M*(*SD*, range), education is formatted as follows: *M* (*SD*, range). Hand refers to dominant hand of the participant throughout their life and is formatted as right: left. Sex is binarised as male or female based on the presentation of the individual at testing as coded as male: female. Missingness information: age (0.49%), education (5.37%), dominant hand (1.46%), sex (0.49%). Due to small number of participants who did not identify as white-British ( n = 7), we coded these as “other” to avoid direct identification, and we do not include ethnicity data with other demographic or performance data.

**Figure 1.**
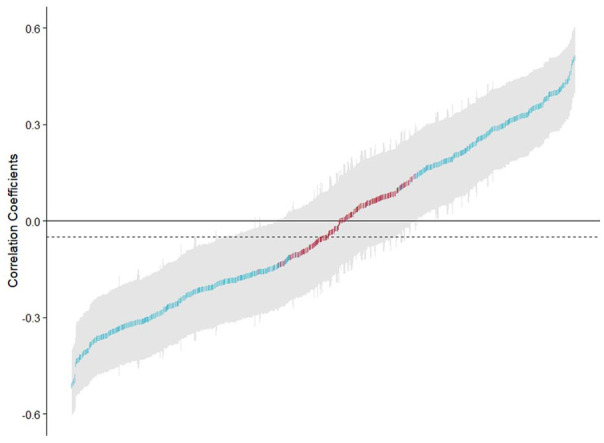
Multiverse Analysis Curve for All Correlations Between the 10 OxMET Metrics, and 30 OCS-Plus Trails and Seven Rule Finding Tasks in a Combined Group Sample of 88 Healthy adults and 117 Stroke Survivors *Note.* The degree of error is presented in light gray at 95% confidence intervals per analysis. In red are non-significant correlations and in blue are significant correlations at the .05 alpha level. The horizontal dotted line represents the median overall correlation coefficient. Figure available at https://osf.io/rv7k8 under a CC-BY4.0 license. OxMET = Oxford Digital Multiple Errands Test; OCS-Plus = Oxford Cognitive Screen–Plus.

Figure 1 illustrates the multiverse for the combined samples group, based on the different specification combinations from Table 1, which generated the different coefficients. The graph illustrates the correlation coefficients found, ordered numerically, and color-coded according to significance (below .05) and the number of individuals included in analysis. A full multiverse analysis curve with graphical presentation of which variable across sample, covariate, and metric type was included can be found in Figure S1 in supplementary materials.

### Sample Group Multiverse Outcomes

**Figure 2.**
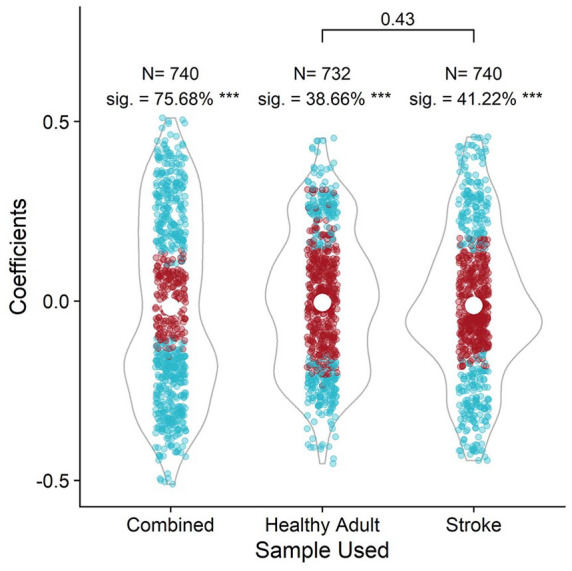
The Distribution of Correlation Coefficients Across Three Sample Groups. *Note. N* refers to the number of correlations computed per sample group, and sig. refers to the percentage of correlations per group that were statistically significant. “***” refers to the significance level (*p* < .002) when comparing the percentage of significant results to the percentage of significant results in 500 iterations of permutated data to create a null effects model. Horizontal bars between groups reflect Welch Two Sample *t*-tests *p* values. Figure available at https://osf.io/mt6xf under a CC-BY4.0 license.

We compared the coefficients obtained from the combined and healthy adults and stroke only samples to their respective 500 permutated null effects models to establish whether the found effects were statistically different to no effect. All sample groups were statistically different to the null effects models at the *p*<.002 level (denoted as “***” in Figure 2). Overall, the sample groups were more robust to deviations in analysis technique than would be expected if the null were true, where the null states that there is no relationship between the variables in the validation correlations. Next, we compared the observed specification curves of the healthy adult, stroke survivor, and combined sample groups to see if there was a significant difference between samples used in the hypothetical validation studies. We compared the found correlation coefficients of the healthy adult and stroke sample groups using Welch Two Sample *t*-tests and found that the groups were not statistically different in correlation coefficients.

### Sample Size Multiverse Outcomes

**Figure 3.**
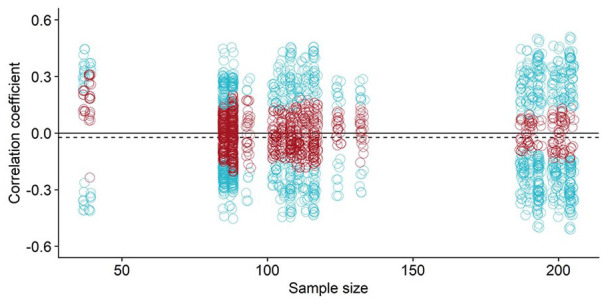
Illustrates the Multiverse Correlation Coefficients Across All Sample Groups Where Coefficients Are Ordered by Number of Observations on the X-Axis. *Note.* The *x*-axis reflects actual sample sizes per correlation. In red are non-significant correlations and in blue are significant correlations at the .05 alpha level. The horizontal dotted line represents the median overall correlation coefficient. To aid in data visualization, the data points are jittered to see overlapping values. There is a gap between 110 and 170 because few analyses had sample sizes between these. Only the specification correlation coefficients are shown rather than which variables go into the curve, as these can be obtained from Figure 1. Figure available at https://osf.io/szwhg under a CC-BY4.0 license.

Next, we examined the impact of sample size on validation outcome, by pooling all analyses together irrespective of sample group. See Figure 3 for an illustration of coefficients by sample size collapsed across all three sample groups. Evidently, we know that the larger the sample the smaller the *p*-value (Thiese et al., 2016), however, the extent of sample size impact on the observed correlation coefficient is not known when only using one sample group in a single study. We found that sample group in this illustration did not visible alter the observed correlations, with similar correlation strengths seen at all sample sizes.

### Validation Measure Choice Multiverse Outcomes

Using only the full combined sample, we examined the impact of comparing metrics that are within and between categories, that is, whether the x variable in a correlation is an accuracy, time, error, or composite/ratio score compared with whether the *y* variable is an accuracy, time, error, or composite/ratio score. Intuitively, one would want to compare within categories, that is, accuracy to accuracy scores and not accuracy to time scores. However, this is not always what is completed in practice. For instance, in a recent validation of the OCS-Plus, some accuracy/error tests were compared with time measures without a clear examination of whether this would affect validation outcomes (Demeyere et al., 2021).

When using a Wilcoxon rank sum test with continuity correction for the difference between the within- and between-category correlations in the multiverse results, we found that the correlation coefficients were statistically different ( W = 23,163, *p*<.001, r = .25 95% CIs for r = [.16, .33]. We examined the specific categories of OxMET variable (accuracy, time, error, or composite/ratio) and the effect of between- or within-group analyses types on coefficient outcomes, where “median between” refers to the specific OxMET variable compared with non-same category variables, and where “median within” refers to specific OxMET variables compared within same category (e.g., comparing accuracy measures to accuracy measures, etc.). See Table 3 for the results of the category comparisons.

**Table 3.** Illustrates the Statistical Significance of Comparisons of Correlations That Are Within- and Between-Category for Individual OxMET Metrics.

OxMET measure	Median between	Median within	Sig.	*r*	*r* 95% CIs
Accuracy	−.05	.38	.017	.28	.03 to .51
Time	−.21	.23	<.001	.53	.39 to .66
Error	−.05	.17	.05	.11	.01 to .24
Composite/ratio	−.18	.29	.009	.31	.07 to .5

*Note.* We used Wilcoxon rank sum with continuity correction comparisons and present *r* as the effect size. Within-category refers to correlations between variables that match in type of metric, such as accuracy-accuracy or error-error metric comparisons, thus between-category comparisons refer to comparisons including accuracy-time, or error-ratio score metric comparisons.

### Covariate Inclusion

Next, we assessed the inclusion of age and education covariates in each analysis through the use of partial correlations with both age and education factored out to see the impact they had on validation outcomes. We compared the combined sample median coefficient without covariates (−.05) and with covariates (−.05) using a Wilcoxon rank sum with continuity and found no significant difference ( W = 68,860, *p*= .89, r =.01, 95% CIs for r = [0, .08]).

## Discussion

The aim of this article was to showcase the effects of sample group, sample size, outcome measure metric choice, and covariate inclusion on validation study outcomes. We used the convergent validation of the OxMET (Webb et al., 2021) to illustrate. A multiverse analysis totaling 2,220 possible analyses was conducted on our data. This was not an exhaustive combination, lending the possibility for many more scoring specifications than those tested in this example (such as analysis technique used, outlier inclusion/exclusion, sample inclusion/exclusion criteria, etc.). The results of the multiverse analysis were in compliment to the original authors’ findings that were largely significant in their anticipated direction in their paper (Webb et al., 2021) and highlight the wider selection of analysis pipelines, the authors could have followed and found very different results.

Using a combined sample of healthy controls and stroke survivors, we found that the choice of the specific metric for a variety of outcome measures in the validation affects the associations of convergent validation outcomes, with some metrics leading to small and non-significant correlations, and others leading to larger significant correlations. However, the largest correlations found in the current study were only just above the benchmark strong correlation in psychology of >.40 (Funder & Ozer, 2019) and the benchmark for convergent validity correlation coefficients >.30 (Rotenberg et al., 2020). Using different sample groups in our analyses did not affect the pattern of observed correlations between outcome measures, meaning that there was little change in convergent validation outcomes when using just healthy controls or just a clinical group.

Furthermore, combining groups led to a greater number of reported significant correlations, likely due to the larger sample size. However, when investigating the different samples per correlation in the multiverse analysis, we found that there was little variance in the observed correlation coefficients, which is counter-intuitive as we would expect greater variance at smaller samples and more stability with larger samples (Schönbrodt & Perugini, 2013; Thiese et al., 2016). We interpret this to mean the associations between outcome metrics were robust. This is further evidenced through our covariate analyses that found no differences when adjusting correlations for age and education or not.

When there are many possible metrics to consider for a single test, turning to the literature is not always the simplest way to pick an outcome measure. Literature on the same task still often operationalizes the metrics differently and there is often no consensus on the best method for deciding the comparison metrics (e.g., the myriad ways of determining trail-making task performance). Authors rarely justify their outcome measure or scoring technique of said outcome measure, making it more difficult for other researchers to decide on their own technique. When a researcher does choose a metric, they may fail to validate their task simply due to the vastly different ways the same test could be scored, given the mixed results of sensitivity of scores (Lange et al., 2005).

All metrics, samples, and comparisons should be justified a priori. The justification is ultimately determined by two points: (a) the purpose of the measure being validated and (b) the form of validation being investigated. We recommend that researchers pre-register or submit their study as a registered report, when undertaking a psychometric investigation, and follow standards of psychometric validation (American Educational Research Association et al., 2014). To determine the best metric to use from a measure, a researcher must first establish exactly what it is they are hoping to validate. For example, if it is a new screen for mild cognitive impairment, they must only select metrics from comparator measures that have been shown to detect subtle cognitive impairments, rather than metrics from the comparator measure which are sensitive to gross-level cognitive decline. In this way, a researcher is able to establish which metric(s) to use in their investigation, based on literature and the theoretically relevant constructs measured by the metrics/measures.

To determine the sample group, again the purpose of the measure must be considered, for instance, including those with dementia and neurologically healthy controls when developing dementia screen with normative data, rather than those with depression alone, etc. There are multiple ways to determine sample size; researchers must consider how many participants are needed to have sufficient power for analyses chosen (Lakens, 2022) but also how representative the samples are needed to be for the purpose of the measure. For example, if a dementia screen is created, the normative sample should reflect the characteristics of the population of interest. We recommend determining how many participants are needed regarding power, and then stratifying the sample to best reflect the population of interest.

Finally, we recommend that when choosing the analyses, again the focus needs to be on the purpose of the measure but also the question being answered in the research. If a new screening tool is created in the format of a bi-dimensional questionnaire, it is critical to establish the factor structure of the questionnaire, as such your analysis technique is pre-determined by the research question. In fitting with sample chosen and sample size, when doing convergent validity analysis, there are several guides for estimating sample size for the common validation techniques, such as factor analysis (Gagne & Hancock, 2006; Wolf et al., 2013), intraclass correlations (Bujang, 2017), and correlations or regressions (Cohen, 1992b). In addition, there are important statistical and theoretical considerations for the inclusion of covariates in convergent validity analysis, see Raab et al. (2000) for a discussion of the impacts and calculations of covariate inclusion.

Although we present the multiverse technique for use in assessing degrees of freedom in convergent validity analysis, the technique can be used for any type of analysis, including factor analysis/latent modeling, or sensitivity/specificity analyses, which are all common convergent analysis techniques (Pawlowski et al., 2013). This multiverse approach has been previously shown to be informative in other neuropsychological research, which used adapted code from the current study (Moore & Demeyere, 2022). Importantly, in addition to a multitude of potential analysis techniques and analytical decisions, core theoretical or measurement models will drive the interpretation of validation research. It remains imperative these frameworks are clearly reported and justified.

### Study Limitations

We note that not all manipulations may be executed by a researcher based on the field norms and standards, and therefore the analyses present in the current study extend beyond the realistic choices. However, each analysis outcome measure or analysis manipulation in this article was chosen based on plausibility. In addition, the sample sizes used in the exemplar are not large enough to detect all potential effects, as correlations tend to stabilize at 250 participants (Pawlowski et al., 2013). We, therefore, encourage caution in interpreting the results of the exemplar by noting that this is an example of a real validation study and not reflective of best practice. Furthermore, while the most common method of assessing convergent validity in Multiple Errands Test literature, correlations are a very basic statistic and perhaps alternative methods such as factor analytical or structural equation modeling may be better suited to assess these relationships.

Foremost, this article aimed to highlight issues in validation studies and to inspire researchers to consider their analytical pathway choices in future research.

## Conclusion

Here, we used a multiverse technique to demonstrate the impact of selecting different plausible outcome variables on convergent validity analysis outcomes in healthy adult and clinical samples. We aimed not to instruct researchers to use the multiverse to bypass non-significant findings in their research but to encourage better practice and full justification of sample size, group, analysis techniques and decisions, etc. as these may all have an impact on the outcomes of validation studies.

We provide a clear example method and instructions for multiverse analysis, inclusive of all code and data to be adapted by future researchers. Avoiding potentially misdiagnosing the nature of any cognitive impairments based on new neuropsychological assessments is paramount, and researchers must ensure tests are robust and generalizable across experimental manipulations and clinical samples.

## Supplemental Material

sj-docx-1-asm-10.1177_10731911221127904 – Supplemental material for Using Multiverse Analysis to Highlight Differences in Convergent Correlation Outcomes Due to Data Analytical and Study Design Choices Click here for additional data file. Supplemental material, sj-docx-1-asm-10.1177_10731911221127904 for Using Multiverse Analysis to Highlight Differences in Convergent Correlation Outcomes Due to Data Analytical and Study Design Choices by Sam S. Webb and Nele Demeyere in Assessment
