# Supplementary material for: Using Multiverse Analysis to Highlight Differences in Convergent Correlation Outcomes Due to Data Analytical and Study Design Choices
Source: Assessment. 2022 Sep 29;30(6):1825–35. doi: 10.1177/10731911221127904 (PMC10363922; doi:10.1177/10731911221127904)
Supplement: sj-docx-1-asm-10.1177_10731911221127904 – Supplemental material for Using Multiverse Analysis to Highlight Differences in Convergent Correlation Outcomes Due to Data Analytical and Study Design Choices [file sj-docx-1-asm-10.1177_10731911221127904.docx]

###
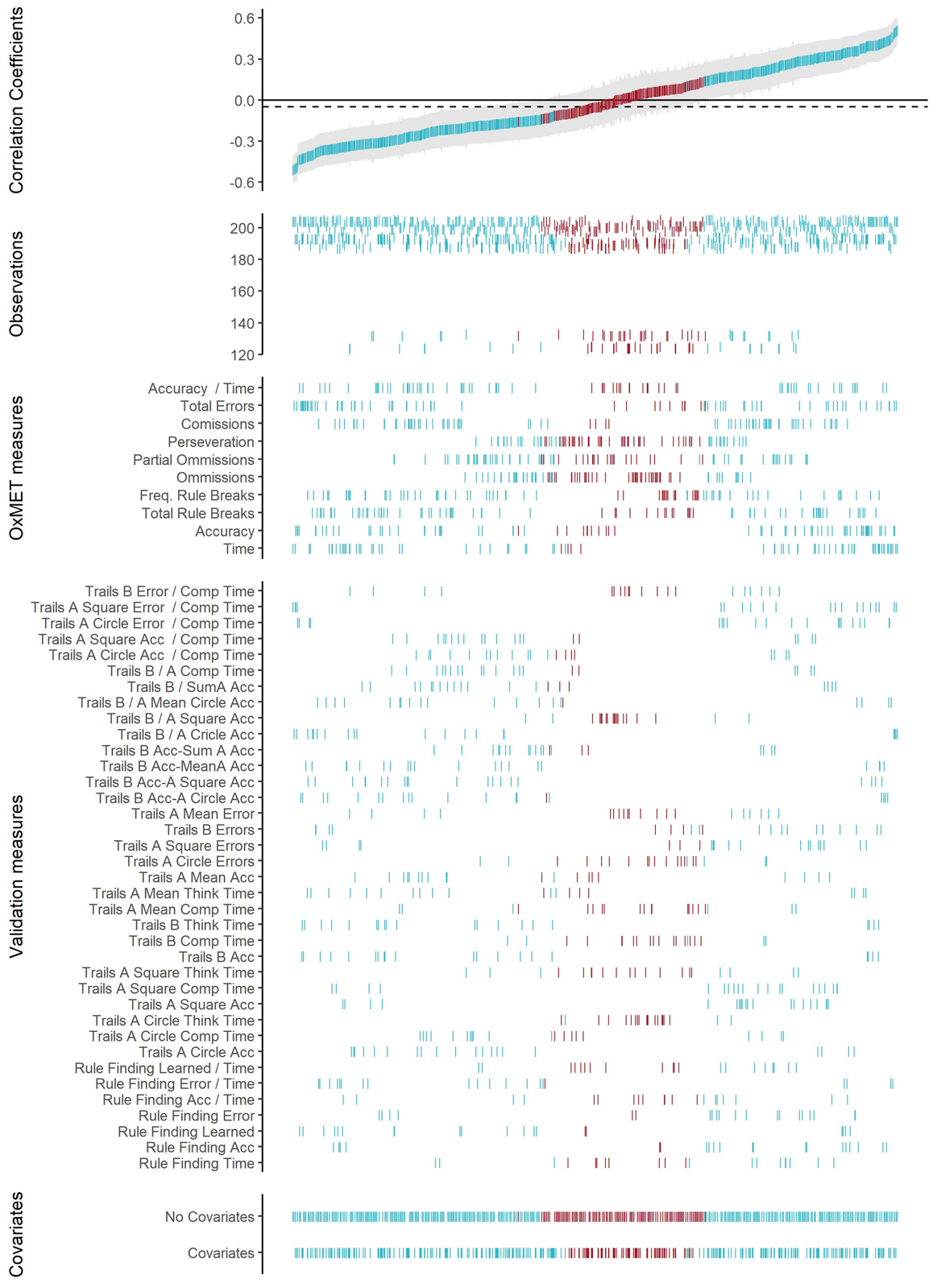
Figure S1. All correlations between the 10 OxMET metrics, and 30 OCS-Plus Trails and seven Rule Finding tasks in a combined group sample of 88 healthy adults and 117 stroke survivors.

*Note*. The degree of error is presented in light grey at 95% confidence intervals per analysis. In red are non-significant correlations and in blue are significant correlations at the .05 alpha level. The horizontal dotted line represents the median overall correlation coefficient. Variables included in each specification are noted by vertical lines in subsequent panels. Figure available at <https://osf.io/7t9ac> under a CC-BY4.0 license.

### Steps for running a multiverse analysis on data already collected.

The following is adapted from Simonsohn et al. (2020), specifically for convergent validity analyses:

To begin a multiverse analysis for a neuropsychological validation, identify any and all theoretically plausible specifications for an analysis in a detailed and logical order. First, determine all potential instances of the dependent or outcome variable of interest. Next, specify all potential instances of the independent variable and potential covariates or confounding variables in the data you have. Variable inclusion can be based on previous studies, or original test guidance, or theoretically or practically plausible metrics. For instance, if one metric is more sensitive to frontal brain damage than others, and this matches the aim of the validation, then include this metric.

Next, specify if there are any different sample criteria. In our case, this was around the decision whether to run separate convergent validation analyses for healthy adults and stroke survivor cohorts or merge the groups for added variance and power. Though sample groups could differ in clinical characteristics, such as the presence of neurological disease, severity, phenotype etc. The impact of these clinical characteristics in outcomes of studies is discussed elsewhere (Moore & Demeyere, 2021) Finally, explicitly determine different decisions to be made about data cleaning including imputation of missing data and removal of outlier data.

When the specifications are mapped out develop a statistical model to run (i.e., correlation or partial correlation, ANOVA, another linear model etc), first create this model to run every possible combination of the specifications (e.g., x-variables x y-variables x covariates x outlier removal x participant group etc = total analyses) in a loop. Existing code for this can be found in the repository for this project (DOI 10.17605/OSF.IO/CKTJ9), and see Orben & Przybylski (2019) for an example of multiverse analysis with associated code. Secondly, in order to compare the specification outcomes, a researcher must compute a null hypothesis curve, that is a curve which reflects the specifications without any true effect, and then run permutations to simulate the variations of null curves (Ferguson & Heene (2012) suggests 500 repetitions). To do this, use the existing statistical model and prior to running the analysis, randomly shuffle the x-variable data (the independent variable in some cases) such that no true relationship exists between x and y variables in the analysis. When this is permutated 500 times, a null specification curve with 500 different outcomes of null effects is created. For example, when comparing a new test to existing tests, shuffle the data in the new test so there is no true correlation between the new and extablished tests. Third, to take inference from the resulting curves, the p-value for significance of a difference between medians from the observed and null effects multiverse is extracted by calculating the proportion of the 500 null analyses which have a test statistic that is the exact same median value or greater as in the observed data. The proportion is divided by two, and the result is significant if below <.05 or if no null values are equal or greater, significance is reported as p <.002. If the outcome is less than .05, then there is a significant difference in coefficients across the observed and null curves supporting the interpretation that the observed data provide robust evidence to reject the null, irrespective of analysis decisions.
